# Considerations of CD8^+^ T Cells for Optimized Vaccine Strategies Against Respiratory Viruses

**DOI:** 10.3389/fimmu.2022.918611

**Published:** 2022-06-15

**Authors:** Toshiro Hirai, Yasuo Yoshioka

**Affiliations:** ^1^ Vaccine Creation Group, BIKEN Innovative Vaccine Research Alliance Laboratories, Institute for Open and Transdisciplinary Research Initiatives, Osaka University, Suita, Japan; ^2^ Vaccine Creation Group, BIKEN Innovative Vaccine Research Alliance Laboratories, Research Institute for Microbial Diseases, Osaka University, Suita, Japan; ^3^ Laboratory of Nano-design for Innovative Drug Development, Graduate School of Pharmaceutical Sciences, Osaka University, Suita, Japan; ^4^ The Research Foundation for Microbial Diseases of Osaka University, Suita, Japan

**Keywords:** aging, attrition, booster vaccines, SARS-CoV-2, tissue-resident memory T cell, CD8 T cells

## Abstract

The primary goal of vaccines that protect against respiratory viruses appears to be the induction of neutralizing antibodies for a long period. Although this goal need not be changed, recent severe acute respiratory syndrome coronavirus 2 (SARS-CoV-2) variants have drawn strong attention to another arm of acquired immunity, CD8^+^ T cells, which are also called killer T cells. Recent evidence accumulated during the coronavirus disease 2019 (COVID-19) pandemic has revealed that even variants of SARS-CoV-2 that escaped from neutralizing-antibodies that were induced by either infection or vaccination could not escape from CD8^+^ T cell-mediated immunity. In addition, although traditional vaccine platforms, such as inactivated virus and subunit vaccines, are less efficient in inducing CD8^+^ T cells, newly introduced platforms for SARS-CoV-2, namely, mRNA and adenoviral vector vaccines, can induce strong CD8^+^ T cell-mediated immunity in addition to inducing neutralizing antibodies. However, CD8^+^ T cells function locally and need to be at the site of infection to control it. To fully utilize the protective performance of CD8^+^ T cells, it would be insufficient to induce only memory cells circulating in blood, using injectable vaccines; mucosal immunization could be required to set up CD8^+^ T cells for the optimal protection. CD8^+^ T cells might also contribute to the pathology of the infection, change their function with age and respond differently to booster vaccines in comparison with antibodies. Herein, we overview cutting-edge ideas on CD8^+^ T cell-mediated immunity that can enable the rational design of vaccines for respiratory viruses.

## Introduction

Vaccines are one of the most important innovations in medical science and public health that can prevent infection and thus reduce the morbidity and mortality caused by infectious diseases (1). Vaccine-based protection against infection can be mediated by two major arms of acquired immunity: antibodies and T cells, which are further categorized into two main subsets: CD4^+^ and CD8^+^ T cells. CD4^+^ T cells, also called helper T cells, coordinate immune responses by promoting antibody responses and CD8^+^ T cells responses. CD8^+^ T cells, also called killer T cells, eliminate intracellular pathogens by directly targeting infected cells. However, their induction by vaccines for respiratory viruses has been less evident than that of antibodies, since sufficient amounts of neutralizing antibodies achieve sterilizing immunity by themselves in many cases. The recent long-lasting coronavirus disease 2019 (COVID-19) pandemic, with the continuous emergence of antibody-escaping virus variants and waning of immunity provided by vaccines, has necessitated improvements in the current vaccine strategy.

Herein, we discuss recent cutting-edge findings related to CD8^+^ T cells that can be better considered for the rational design of more-effective and safer vaccines against acute respiratory infections. We begin by describing the role of CD8^+^ T cells in protection and their potential pathological role in respiratory infection. We then detail several considerations for better vaccines: changes in T cell responses with age; the importance of localized CD8^+^ T cells for their optimal protective function; and possible concerns about repeated boosters with regard to CD8^+^ T cell immune memory, such as competition among memory compartments. The focus of this review is to overview recent concepts related to CD8^+^ T cells, and we have intentionally omitted a discussion on T cell markers and the molecular mechanism of their induction/maintenance to appeal to a broader audience.

## Beneficial Role of CD8^+^ T Cells in Acute Viral Infection

CD8^+^ T cells have been extensively shown to promote the clearance of viruses, including influenza virus, respiratory syncytial virus and coronavirus, in mouse models. In many cases, however, either the models used are antibody-incompetent or the vaccines used only induce CD8^+^ T cells to challenge the viruses (2, 3). On the other hand, there are many reports showing that depletion of CD8^+^ T cells has little effect on vaccine efficacy in antibody-competent hosts, which appears to be a more common situation in real life (2). Then, can the induction of CD8^+^ T cells add value to vaccines that can induce antibodies?

Firstly, a “CD8^+^ T cells only” situation is not so uncommon. CD8^+^ T cells develop earlier than antibodies after prime vaccination with a severe acute respiratory syndrome coronavirus 2 (SARS-CoV-2) spike protein mRNA vaccine and thus appear to be responsible for the vaccine-mediated protection in the early time window (4). Exposure to SARS-CoV-2 can induce virus-specific T-cell responses without seroconversion (5), which suggests that the antigen requirement to initiate cellular immunity is lower than that for humoral immunity. There are also many people who have impaired ability to mount cellular or humoral immune responses to vaccination for genetic cancer-related reasons and the immunosuppressive drug regimens associated with these (e.g., X-linked agammaglobulinemia, leukemia or lymphoma and their treatments, i.e., anti-CD20 or CAR-T cell therapies) and they hence constitute a high-risk group for respiratory infection with severe disease, including COVID-19 (6–12).

Then, if we have vaccines to induce strong neutralizing-antibody responses, do we still need CD8^+^ T cells? Given the wide variety of genetics and environmental differences, it is never true that all vaccinees will respond to a vaccine with the same magnitude and that everyone would achieve and maintain a sufficient level of neutralizing-antibodies that completely controls viruses. A study in convalescent rhesus macaques in which CD8^+^ T cells were depleted showed that CD8^+^ T cells can protect against rechallenge with SARS-CoV-2 in the setting of waning and sub-protective antibody titers (13). Furthermore, T cell-inducing vaccines durably prevent mucosal simian–human immunodeficiency virus (SHIV) infection even with low neutralizing-antibody titers (14). CD8^+^ T cells can work as a backup for people who fail to induce sufficiently high levels of neutralizing-antibodies and those who once had a high titer of antibodies but lost it over time.

When considering viral escape from host immune systems by mutation (i.e., antigenic drift), vaccines that induce CD8^+^ T cells are more likely to remain protective. Viral escape from T cells, particularly for viruses that induce acute infection that requires rapid transmission to individuals, is less of a concern because viral escape from T cells restricted by just a single set of major histocompatibility complex (MHC) molecules in an individual does not effectively spread in a genetically diverse population, owing to the remarkable polymorphism of the MHC (15).

A recent study of the Omicron SARS-CoV-2 variant has shown that antibodies induced by infection with previous variants or by mRNA vaccines (e.g., BNT162b2) inhibit the entry of the Omicron variant 80-times or 34-times less efficiently, respectively, compared with those induced by the B.1 variant spike (16, 17). In addition, the Omicron variant evades neutralization by most therapeutic monoclonal antibodies (18). T-cell reactivity to the Omicron variant, however, has been reported to be preserved in most individuals who were previously infected with another SARS-CoV-2 variant or had been vaccinated; hence, T cells can cross-recognize variants, from Alpha to Omicron (19–21). It has even been reported that the magnitude of T cells that are cross-reactive to Omicron is similar to that for Beta and Delta variants, despite Omicron harboring considerably more mutations that enable escape from antibodies (22).

Influenza virus is another representative virus that escapes from antibodies *via* mutations. Notably, CD8^+^ T cells induced by seasonal influenza can cross-recognize the 2009 pandemic H1N1 virus (23, 24) and the pre-pandemic avian influenza A (H5N1) virus (25, 26), both of which resulted from antigenic shifts. Importantly, higher ratios of pre-existing T cells to conserved CD8^+^ T cell epitopes were found in individuals who developed less-severe illness due to the H1N1 virus (27), consistent with an observation that individuals unexposed to SARS-CoV-2 but having CD8^+^ T cells specific to conserved epitopes among seasonal human coronaviruses tend to show mild illness (28). On the other hand, non-neutralizing antibodies and CD8^+^ T cells are not independent effectors but might work in synergy against heterosubtypic influenza infection (29), supporting the idea that the induction of both antibodies and CD8^+^ T cells ensures a long-lasting protective effect even against frequently mutating viruses.

Recent studies suggest that IFN-γ production from CD8^+^ T cells enhances cellular and humoral immune responses following immunization (30, 31), and that CD8^+^ T cells can also promote proper resolution of inflammation (32). Thus, the induction of CD8^+^ T cells can improve the efficacy of vaccines beyond just killing viruses.

## CD8^+^ T Cells Might Occasionally Contribute to Pathology Associated With Respiratory Infection

Despite their beneficial role in mediating viral clearance, CD8^+^ T cells might promote immunopathology in some situations. This phenomenon is well recognized in the mouse model of respiratory syncytial virus (RSV) infection; it has also been observed in several other virus-infection models, including influenza virus (33–36) and murine adenovirus models (37).

Mice depleted of CD8^+^ T cells exhibit elevated lung viral titers, but weight loss and worsen symptoms of illness following acute primary RSV infection (38). Furthermore, memory CD8^+^ T cells induced by neonatal RSV infection or prime–boost vaccination of a CD8^+^ T cell epitope of RSV promote RSV clearance upon challenge, but significantly exacerbate weight loss and pulmonary pathology (39, 40). A study showed that the transfer of higher numbers of RSV-specific CD8^+^ T cells results in more-severe disease although viral clearance correlates with the number of CD8^+^ T cells (41), consistent with another report showing that high numbers of adoptively transferred transgenic T cells induce protection following low-dose viral challenge of influenza virus but exacerbate infection after high-dose challenge (33). Thus, strong CD8^+^ T cell responses unaccompanied by optimal CD4^+^ T cell-mediated or antibody-mediated responses, and hence not regulated appropriately, might potentially result in exacerbated pathology of acute respiratory infection. In addition, we would note that another hypothesis is raised by a recent study that some specific types of CD8^+^ T cell (e.g. IL-4 secreting CD8^+^ T cells) might cause immunopathology (42). Together, there might be specific situations wherein CD8^+^ T cells are more harmful than beneficial in controlling respiratory infection. Further studies are necessary to elucidate the context and mechanism.

Nevertheless, infants who are fatally infected with influenza virus or RSV show few CD8^+^ T cells in the lung regions (43, 44). Additionally, infants who are severely infected with RSV exhibit low expression of genes related to CD8^+^ T cell responses (45). In support of a protective, rather than pathogenic, role for CD8^+^ T cells, correlations between increased CD8^+^ T cell cytolytic activity and cytokine production with reduced symptom scores, faster recovery and fewer fatalities following H1N1 or H7N9 influenza virus infections have been identified (46, 47). Further studies are required to determine the situations wherein CD8^+^ T cells contribute to pathology, for safer development of CD8^+^ T cell vaccines.

## Age-Associated Issues in Inducing CD8^+^ T Cells

One of the most significant risk factors of severe illness due to acute respiratory infection is aging. It is well recognized that the majority of deaths from seasonal influenza occurring among the elderly correlate with age after adulthood (48, 49). The recent SARS-CoV-2 pandemic has confirmed the susceptibility of the elderly (50); hence, the elderly population requires vaccines the most. Among the anatomical and physiological changes during aging that may be associated with the susceptibility to infection, changes in acquired immune responses are notable (51). Many studies have shown poorly induced immune responses to vaccines, in terms of both cellular and humoral immunity, among the elderly (52–54), whereas many healthy elderly individuals exhibit sufficient protective responses to vaccines (55).

Responsiveness to vaccines can be determined using the frequencies of naive T cells that can recognize epitope peptides from the vaccines (56). Therefore, the T cell population has to maintain a highly diverse set of specificities in sufficient frequencies or lose the ability to respond to a vaccine for protection. T cells develop in the thymus, and thymic activity appears to be the key to maintain the diversity of naive T cells. The production of new naive T cells from the thymus, however, rapidly decreases after infancy and there is an established reduction in thymic function during puberty (57, 58). Thymic output further declines sharply after 40 years of age, although residual thymic activity is observed (59, 60). However, even after this age, naive T cells are functionally maintained predominantly in lymph nodes (60).

The major factor that maintains functional naive T cell populations is homeostatic proliferation throughout life rather than thymic output, and the decline in thymic function can be compensated by minimal adjustments in such proliferation (56). It has been reported that thymectomy in adult humans did not increase the incidence of infections for several decades; there were no significant changes in naive T cell numbers, in contrast to the effect of neonatal thymectomy (61–63). However, vaccine efficacy in older populations has often been observed to be low (53, 54).

In addition, irrespective of the mechanism of homeostatic proliferation, a preferential decline in T cell reactivity to viral epitopes occurs if there is a low frequency of naive precursors that recognize those epitopes, in some cases leading to “holes” in the T cell repertoire (64). A potential environmental factor that induces such a discrepancy is cytomegalovirus infection, the seroprevalence of which approaches 80% by the age of 70 in northern Europe; several studies have shown that cytomegalovirus seropositivity correlates with reduced efficacy of vaccines in the elderly (65). The infection induces significant expansion of cytomegalovirus-specific CD8^+^ T cell populations, which is called “inflation,” and the unique immune responses reduce the diversity of the naive and memory T cell pool, potentially because of competition among T cells for finite resources (65). There are numerous environmental factors that might disrupt homeostatic maintenance of the T cell pool that are yet to be determined.

In addition to the reduced diversity of T cell populations, cellular senescence of T cells is a potential reason for defects in the ability to respond to vaccines. It is well recognized that CD8^+^ T cell populations show more-evident senescent phenotype characteristics, such as loss of self-renewal ability and the accumulation of differentiated dysfunctional cells, than CD4^+^ T cells do (66, 67). Telomere shortening and DNA damage resulting from replicative stress induced by homeostatic turnover might cause cellular senescence in T cells and many other types of cells; the characterization of cell-intrinsic drivers of T cell aging is being actively studied (68).

Notably, T cell senescence not only appears to reduce the ability to form efficient protective memory but also induces dysfunctional memory cells that might lead to a pathological effect. A recent study showed that influenza infection caused chronic unresolved lung pathology in old mice but not young mice (69). Although the magnitude of the systemic CD8^+^ T cell response was significantly low in old mice, notable accumulation of dysfunctional and unprotective CD8^+^ T cells was observed in the lungs and the depletion of these cells rescued the old mice from the lung pathology following influenza infection.

The phenotype of CD8^+^ T cell accumulation in peripheral tissues has also been observed in old naive mice (70). One month of exposure to the environment in old mice sufficiently converts adoptively transferred young donor CD8^+^ T cells into the aged phenotype, which is characterized by the expression of exhaustion markers and abnormal effector responses to T cell antigen-receptor stimulation in addition to accumulation in various peripheral organs (70). Notably, the phenotype may be irreversible because it persists even after 1 month of exposure to the environment in a new, young host. Thus, aged environments are responsible for the dysfunctional, possibly pathological, phenotype.

Age-related changes are important considerations for vaccines not only for the elderly but also for the young generation. Newborn infants appear unable to induce strong IFN-γ-mediated type-1 immunity, possibly because of both cell-intrinsic and cell-extrinsic reasons (71–73). It has been reported that infant mice with influenza infection generate markedly fewer memory CD8^+^ T cells in the lungs than adult mice do, resulting in less control of a heterosubtypic virus following a secondary challenge (74). The reduction in the number of memory cells induced in the lungs is due to a cell-intrinsic reason, as adoptively transferred CD8^+^ T cells from infants fail to populate the lungs. Sparse memory cells in infant lungs and enriched dysfunctional T cells in old lungs following influenza infection as mentioned above appear contradictory (69, 74). Hence, an effective pan-CD8^+^ T cell vaccine for all age groups might be a challenge, whereas a life stage-specific strategy may maximize the protective efficacy of vaccines.

## CD8^+^ T Cells Function Locally and Their Location Is Key for Protection

Our knowledge of human T cell responses has been extensively derived from analysis of peripheral blood, in which only about 2% of T cells in the whole body exist (75). The majority of T cells are found within lymphoid tissues (bone marrow, spleen, tonsils and an estimated 500–700 lymph nodes) and a big population was recently found in non-lymphoid peripheral tissues, including barrier sites such as the upper and lower respiratory tract, lungs, gut and skin (76).

Importantly, it is the nature of CD8^+^ T cells to localize at sites of infection to exert protective functions that require direct cell contact with infected cells, in contrast to B cell humoral immunity mediated by widespread tissue access to antibodies (77). Although memory CD8^+^ T cells can proliferate faster and fire more rapidly than naive cells upon recognition of specific antigens, better protection by CD8^+^ T cells is supported by better surveillance systems that allow these cells to detect pathogen invasion faster and thus control it better (78). Until recently, it was understood that such surveillance is achieved *via* a re-built T cell recirculation system; some memory cells (i.e., effector memory cells) recirculate through non-lymphoid tissues, sometimes with bias through previously infected barrier sites, wherein naive cells do not seek antigens, whereas some memory cells (i.e., central memory cells), as well as naive cells, are trafficked through lymphoid tissues.

However, it was recently revealed that T cells use an alternative strategy that probably better contributes to protection, namely, memory T cells are embedded into tissues, particularly the barrier sites the pathogen initially invaded. These memory cells are called resident memory T (Trm) cells and, contrary to previous understanding, it is currently known that most tissue-biased surveillance of T cells is achieved by Trm cells that do not return to the blood circulation, by definition (there are exceptions, as mentioned below), and that there are numerically quite rare populations of effector memory T cells that recirculate between tissues and blood (79). Sufficient numbers of Trm cells have the potential to provide near-sterilizing immunity (80, 81). Furthermore, the heterosubtypic protective immunity following influenza virus infection or live-attenuated influenza virus vaccination can be mostly mediated by Trm cells and is independent of circulating T cells and neutralizing-antibodies (82, 83).

Even new vaccines such as CD8^+^ T cell-inducing mRNA and adenoviral-vector injectable vaccines do not induce the most protective Trm cell populations at the respiratory barrier sites the virus would invade, and hence do not fully utilize the high protective potential of CD8^+^ T cells (30). Induction of Trm cells requires the recruitment of effector T cells that are activated in lymph nodes by specific antigens to the peripheral site. Infection induces inflammation at the infected site and causes the recruitment of effector cells that differentiate into Trm cells. Although Trm cells might re-encounter their specific antigens at the non-lymphoid peripheral site, such secondary recognition is not always necessary for Trm cell differentiation; therefore, the application of non-inflammatory chemokines to a T cell-primed host would sufficiently induce Trm cells (84).

**Figure 1:**
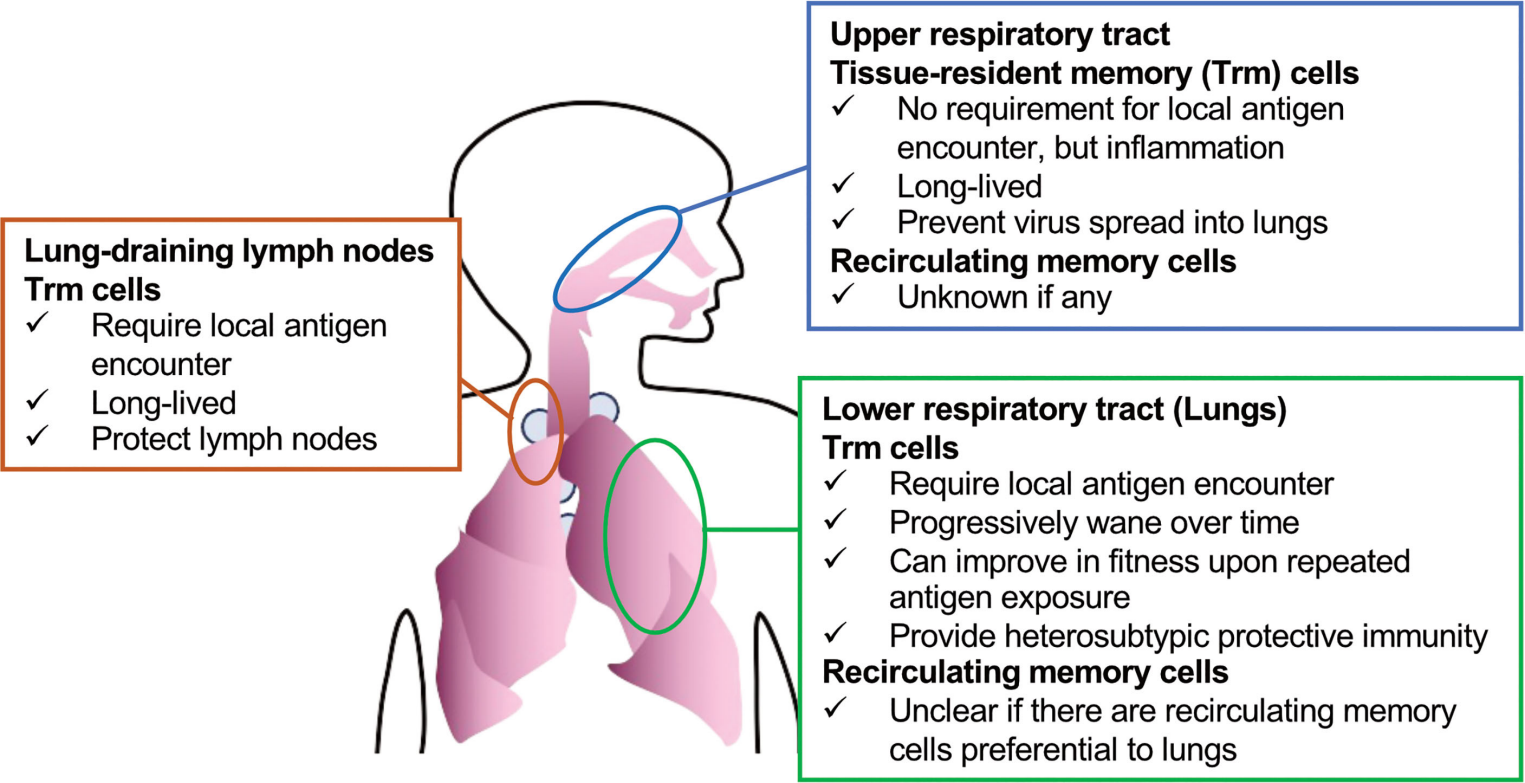
Subsets of CD8^+^ T cells (Trm cells and recirculating memory cells) that can be targeted to protect against respiratory viruses.

However, in certain tissues, including the lungs, secondary antigen recognition at the non-lymphoid tissues appears to be required for Trm cell differentiation (85, 86) (**Figure 1**). Hence, inhaled vaccines may be necessary to induce Trm cells in the lungs. Another challenge for the induction of lung Trm cells by vaccines is that these cells progressively wane over time as soon as the local antigen disappears, potentially due to the specific harsh environment (87–89). This is in contrast to the general Trm cell populations in other tissues that show longevity. Memory T cells circulating in blood can form Trm cells, as well as effector T cells, upon tissue inflammation or antigen recognition (90), and circulating memory cells that experience four repeated antigen recognitions have been shown to form lung Trm cells that exhibit extended longevity (91). Similar enhanced fitness was observed in Trm cells derived from circulating memory cells in ear skin, wherein a few Trm cells derived from primary effectors can be maintained (92, 93). Whether differentiated lung Trm cells can further enhance their fitness by repeated local antigen encounters is to be determined, boosting inhaled vaccines that supply antigens to the lower respiratory tract is a promising way to induce durable lung Trm cells.

Although Trm cells in the lungs rapidly decrease in number over time, they egress to the draining lymph node *via* lymphatic vessels, *via* a process called retrograde migration, and then become Trm cells in the lymph node (94). This might be a lung-specific phenomenon, since draining lymph nodes of skin or the female reproductive tract, wherein Trm cells persist stably, do not induce such cells unless the Trm cells re-encounter their specific antigens and migrate out of the tissue (95). A recent study showed that Trm cells contribute to local lymph node protection; however, the protective role of Trm cells in lymph nodes for upstream barrier sites is to be investigated (96). It is important to remember that antigen administration to the lungs is required for such lymph node-resident Trm cells (94).

Strong immune surveillance of the lungs by CD8^+^ T cells might also be achieved by inducing recirculating memory cells. There are extensive descriptions of gut-homing or skin-homing T cells, which are assumed to have preferential gut-homing or skin-homing capacity (97, 98). It is less clear, however, whether homing T cells that are preferential to the lungs exist, although effector and memory T cells recirculate in these organs (74, 99). It is also notable that maintenance of recirculating memory cells can be affected by the microenvironment in the peripheral tissue to which they recirculate (100), as in the case of the lungs, wherein, at the minimum, Trm cells are poorly maintained. Therefore, even if there are memory cells that recirculate preferentially to the lungs, their longevity needs to be investigated. On the other hand, it has been suggested that secondary memory cells that re-differentiate from Trm cells retain their preferential homing capacity for the tissues wherein the Trm cells had resided (101). Hence, boosting vaccines could also enhance memory cells that recirculate in the lungs.

**Figure 2:**
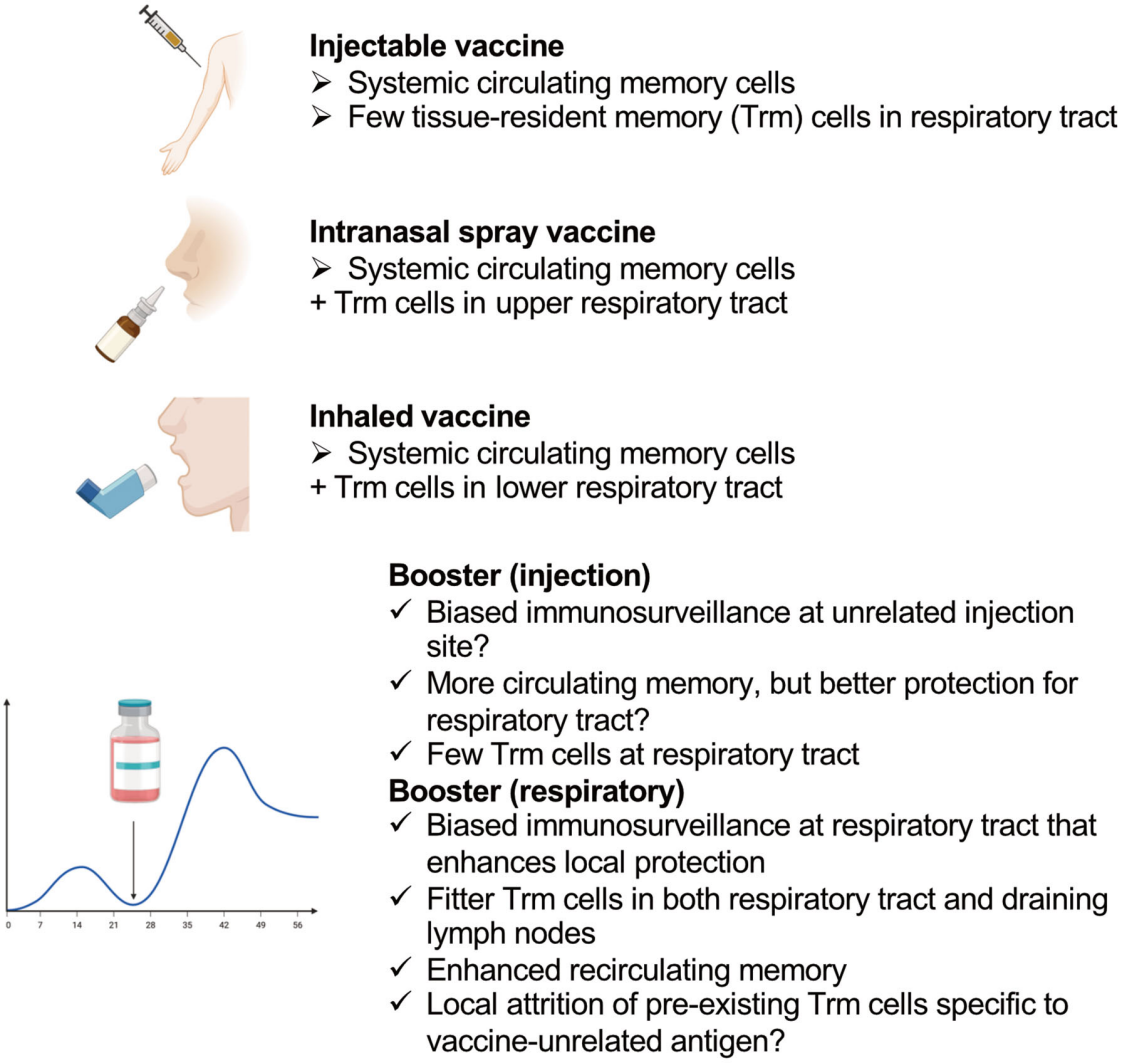
Features of different types of vaccines that induce CD8^+^ T cells and the effects of different locations of boosters.

One licensed live-attenuated influenza virus vaccine (FluMist^®^) comes in the form of a nasal spray; it mainly immunizes the upper respiratory tract rather than the lower respiratory spaces, which include the lungs (102) (**Figure 2**). Natural infections with seasonal influenza A viruses tend to be initiated and localized at the upper respiratory tract (103). Hence, the upper respiratory tract is an important site for the initiation of infection, which might spread to the lungs to induce pneumonia and/or the transmission of the virus *via* coughing, sneezing or talking (104).

CD8^+^ Trm cells induced in the upper respiratory tract have been shown to prevent virus spread into the lungs (105). In contrast to lung Trm cells, they do not require local cognate antigens, and persist stably for a long time (105). Nasal-associated lymphoid tissue is the main target lymphoid tissue of nasal vaccines to induce immunity; however, they are not routinely surveyed by circulating T cells under the steady state, and specific inflammatory stimuli are required to induce immunity in these tissues (106, 107). Numerous ongoing pre-clinical studies are investigating the effects of “nasal vaccines;” however, one potential problem in interpreting the results for human vaccines is that most of these studies are not analyzing the effects of the vaccines on the upper and lower respiratory tracts separately. Human nasal spray vaccines mainly target the nasal cavity, and inhaled aerosolized vaccines target the lower respiratory tract, whereas these pre-clinical “nasal vaccines” can immunize the whole respiratory tract owing to the high volume of the administered vaccine solutions.

An example of the high-volume effect is as follows. The PR8 strain of influenza virus does not spread efficiently from the nasal tissue to lungs in mice (108); a 10 μl volume of infecting PR8 administered to mice *via* the nares showed only limited replication in the lungs, whereas a 30 μl volume showed high titers (105). This might have been because the nasal cavity volume of a mouse is only 32 mm^3^ (= 32 μl) (109). Considering the different roles of Trm cells in the upper and lower respiratory tracts, distinguishing the type of respiratory vaccines in pre-clinical studies may produce better knowledge for human vaccine development.

## Potential Concerns About Repeating Boosters

Boost immunization might expand the populations of Trm cells and Trm cell-derived memory cells recirculating in tissue, as well as draining lymph node Trm cells, resulting in biased immune surveillance of the respiratory tract at the immunized site, as mentioned above. Thus, boosting at the site where the virus would invade is ideal, whereas biased immune surveillance of tissues other than the entry site of the virus (it is likely that a muscle is the injection site) might not be optimal for protection against a respiratory virus.

Although the number of memory cells circulating in blood can be increased by boost immunization, this strategy would not necessarily enhance protection in the lungs, since only specific populations of memory CD8^+^ T cells can protect these organs (110); furthermore, excessive expansion of non-protective CD8^+^ T cell populations might result in pathology, as discussed above. Live-attenuated virus vaccines and viral vector vaccines show promise in inducing CD8^+^ T cells at mucosal sites; however, boost immunization with such vaccines may be less efficient in inducing immunity than primary immunization or previous natural exposure to the virus (111, 112). Heterologous prime–boost vaccination might be a solution when there is a challenge with regard to the cost (113).

Another potential issue of repeated boosters is that they might cause attrition of pre-existing immune memory to vaccine-unrelated, previously encountered pathogens. A loss of pre-existing lymphocytic choriomeningitis virus-specific CD8^+^ memory T cells has been observed following a series of different viral infections (114). Type-I interferon induced by viral infection might be responsible for the loss, since it may induce apoptosis in pre-existing memory cells (115).

The high immunogenicity of mRNA vaccines is exerted *via* innate type I interferon responses (30), and it might be a concern that the activation of innate immunity by the vaccine directly causes the attrition of pre-existing memory cells. Attrition can also be potentially induced by cell competition for finite resources among memory cells. Expansion of monoclonal T cell populations *via* a heterologous prime–boost strategy, however, showed that the expansion of single-epitope-specific T cell populations did not affect the number of pre-existing memory CD8^+^ T cells in the blood and spleen (116). Moreover, the effect can be different in the microenvironment of peripheral tissues. We observed that competition between newly recruited effectors and memory T cells for a tissue signal required for Trm cell persistence could decrease the number of pre-existing Trm cells in the skin epidermis; this suggests that the epidermis is an environmental niche capable of maintaining a finite number of Trm cells (93).

In support of our observation, mice with known-specificity Trm cells under a specific-pathogen-free clean environment significantly lost gut Trm cells following exposure to a dirty real environment with random pathogens, and the gut showed massive infiltration of CD8^+^ T cells with other specificities (117). This phenomenon may have involved local memory attrition due to repeated mucosal immunization. Some specific viral infections, namely cytomegalovirus infections, which are accompanied by inflated CD8^+^ T cell responses, are also suggested to contribute to attrition of immune memory to other antignes (118). Importantly, immunization with an adenovirus vector, which can also induce the inflation of CD8^+^ T cell responses, was reported to not alter the size of the pre-existing memory cell populations (119). Therefore, it is not clear if and how memory T cell attrition can be induced in humans; however, attention should be paid to the potential negative impacts of booster vaccines to fully enjoy the benefits of vaccination.

## Conclusions

SARS-CoV-2 variants have necessitated repeat booster shots of vaccines for two reasons: to induce more-mature antibodies that can neutralize variants; and to recover the immunity against SARS-CoV-2 induced by previous shots of the vaccine that has waned over time. To achieve the first goal, enhancing CD8^+^ T cells, particularly with specificity, which can provide heterosubtypic protection, rather than spike protein-induced antibodies, is a reasonable approach; a vaccine that can induce both CD8^+^ and CD4^+^ T cells for several viral proteins, including nucleoprotein, appears to be very promising (120). On the other hand, antibodies induced by a booster of SARS-CoV-2 mRNA vaccine decline similarly after the secondary dose (121). However, CD8^+^ T cell-based immunity might last longer than that provided by antibodies following SARS-CoV-2 infection (122, 123). Since the infection does induce localization of CD8^+^ T cells to respiratory tissues, it might support an idea that nonlymphoid organs provide a flexible reservoir for the long-term preservation of T cell-mediated immunity (117). Several adenoviral vector-based intranasal and inhaled vaccines for SARS-CoV-2 are under development (124, 125), and we should be looking forward to observing the impact of localized CD8^+^ T cells on virus variants, as well as the extended longevity of T cell memory. Next-generation vaccines equipped to induce an ideal CD8^+^ T cell-based immunity can further extend the successful history of vaccination.

## Author Contributions

TH wrote the manuscript and YY edited it. All authors approved the submitted version.

## Funding

This work was supported by Grants from the Japan Society for the Promotion of Science (JSPS KAKENHI Grant Number JP 21K15468 to TH) and the Takeda Science Foundation.

## Conflict of Interest

YY is employed by the Research Foundation for Microbial Diseases of Osaka University.

The remaining author declares that the research was conducted in the absence of any commercial or financial relationships that could be construed as a potential conflict of interest.

## Publisher’s Note

All claims expressed in this article are solely those of the authors and do not necessarily represent those of their affiliated organizations, or those of the publisher, the editors and the reviewers. Any product that may be evaluated in this article, or claim that may be made by its manufacturer, is not guaranteed or endorsed by the publisher.
